# Type V secretion: mechanism(s) of autotransport through the bacterial outer membrane

**DOI:** 10.1098/rstb.2011.0208

**Published:** 2012-04-19

**Authors:** Jack C. Leo, Iwan Grin, Dirk Linke

**Affiliations:** Max Planck Institute for Developmental Biology, Spemannstrasse 35, 72076 Tübingen, Germany

**Keywords:** autotransport, protein secretion, outer membrane, Bam complex, adhesin, bacterial cell surface

## Abstract

Autotransport in Gram-negative bacteria denotes the ability of surface-localized proteins to cross the outer membrane (OM) autonomously. Autotransporters perform this task with the help of a β-barrel transmembrane domain localized in the OM. Different classes of autotransporters have been investigated in detail in recent years; classical monomeric but also trimeric autotransporters comprise many important bacterial virulence factors. So do the two-partner secretion systems, which are a special case as the transported protein resides on a different polypeptide chain than the transporter. Despite the great interest in these proteins, the exact mechanism of the transport process remains elusive. Moreover, different periplasmic and OM factors have been identified that play a role in the translocation, making the term ‘autotransport’ debatable. In this review, we compile the wealth of details known on the mechanism of single autotransporters from different classes and organisms, and put them into a bigger perspective. We also discuss recently discovered or rediscovered classes of autotransporters.

## 1. Introduction

Gram-negative bacteria display a multitude of proteins on their cell surface. These proteins must cross two membrane systems: the inner membrane (IM)—composed of diglyceride phospho- and glycolipids—and the outer membrane (OM)—which is an asymmetric assembly of inner leaflet phospholipids and outer leaflet lipopolysaccharides. Different protein translocation systems exist in the IM that have been studied in much detail; these are the general secretion (Sec) system with its accessory factors, and the twin arginine translocation (Tat) system. Both operate by recognizing specific signal peptides at the N-terminus of transport substrates, which are cleaved during translocation through the IM [1]. The major difference between the systems is that the Sec system translocates unfolded polypeptide chains, while the Tat system transports folded proteins through the membrane, mostly proteins with cofactors that could not be assembled in the periplasm [2,3]. The Sec system is also the major system for the insertion of helical membrane proteins into the IM [1,4,5]. In this case, no signal peptide is cleaved; recognition of the first hydrophobic α-helix is sufficient for membrane insertion. It is possible that this recognition already happens at the level of mRNA, as hydrophobic residues are coded by U-rich codons [6].

Numerous highly specialized protein secretion systems exist, most being large protein complexes. These systems have traditionally been labelled using roman numbers; today, we recognize secretion systems type I through type VIII, and additionally, the chaperone-usher (CU) system that is used for pilus assembly on the Gram-negative bacterial cell surface [7]. Type I secretion systems are composed of an OM pore component coupled to an IM ABC transporter [8]; here, specific substrates are recognized by a C-terminal, non-cleavable motif and pass both membranes in one step. Type II–IV secretion systems are much more complex structures, composed of many different subunits. They form needle-like or pilus-like structures on the cell surface, which are used to secrete proteins (or sometimes, DNA or protein–DNA complexes in the case of type IV secretion systems) either into the medium (type II) or directly from the bacterial cytoplasm into host cells (types III and IV) [9–11]. The type III secretion system is evolutionarily related to the flagellar apparatus. Type V secretion systems are the autotransporters which will be discussed in detail in this review. Type VI secretion systems constitute a large mechanical cell puncturing device, with similarities to phage injection machineries [12], while type VII secretion systems are limited to mycobacteria, actinobacteria and Gram-positive bacteria [13], which are not discussed here. Type VIII secretion denotes the specific transport system for bacterial curli [14], amyloid-like adhesin structures on the cell surface [15]. The CU system and the type II, V and VIII systems require the Sec machinery for their function; the export of substrates in all other systems is Sec-independent as they completely span both membranes. The Sec-dependent systems do not use adenosine triphosphate (ATP) or a proton gradient for translocation across the OM [16]—this autonomy from cytosolic energy sources is the basis for our use of the term ‘autotransporter’. This extends the original definition of Meyer *et al.* [17] of the transport function and the passenger domain residing in the same polypeptide chain to include type Vb secretion.

**Figure 1.**
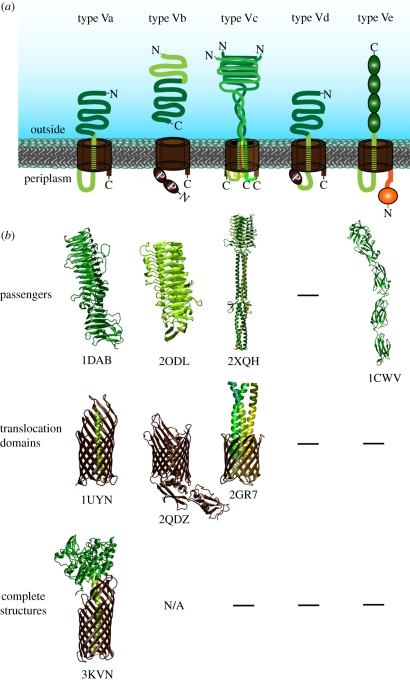
Structures and topology models. (*a*) Topology models of the different type V secretion systems. The translocation domain is displayed in brown, linker/Tps regions in light green, passenger domains in dark green and periplasmic domains in orange. POTRA domains are labelled (P). For clarification of the topology, N- and C-termini are indicated. (*b*) Structural information for passenger domains and transmembrane domains of the different systems is taken from the Protein Data Bank (with PDB codes under each structure). Note that only single examples are displayed and that in some cases, more structures are available; vice versa, for some domains, no detailed structural information is available of any exemplar.

In this review, we compile and discuss the current knowledge on type V secretion. Autotransporters come in different flavours, and are classified into monomeric and trimeric autotransporters, and two-partner secretion systems (TPSS). The common principle of all autotransporters is their dependency on the Sec machinery for IM transit, and the presence of a β-barrel domain that inserts into the bacterial OM, where it acts as a transporter for the so-called passenger domain(s) destined for surface localization. Figure 1 shows the different structures known for the transmembrane parts of different type V secretion systems, and includes topology models for recently described types where no structural information is available yet. In this review, we will describe the different subtypes of autotransporters in detail and conclude with the common mechanism that underlies type V secretion.

## 2. A note on nomenclature

Autotransporters are defined by their transmembrane domain located in the Gram-negative OM. This transport domain is sometimes referred to as the translocator domain, β-domain or the helper domain. The domain that is being translocated (or helped) is typically called the passenger domain, or is referred to as the extracellular domain or α-domain. Note that most autotransporters export numerous domains to the cell surface, leading to multi-domain passenger ‘domains’. The diversity of exported domains is very large, and thus, classification is done according to the transport domain.

The term ‘autotransport’ was first used by Thomas Meyer and colleagues, who investigated IgA protease from *Neisseria meningitidis* [17,18], based on the earlier finding that the polypeptide chain of the protein itself hosts the function for surface display, and thus for translocation across the OM. The term ‘autodisplay’ is used synonymously, especially in biotechnology, where heterologous proteins are fused to the autotransporter for surface expression [19,20]. The nomenclature of type V secretion for autotransporters was introduced by Henderson *et al.* [21], after confusion with type IV secretion systems, and a systematic classification into type Va to type Vc autotransporters was published in 2004 [22]. We kept the latter nomenclature for the organization of this manuscript, adding two more (sub-)classes (types Vd and Ve) from recent literature and our own findings.

## 3. Type Va secretion: classical autotransport

Monomeric autotransporters were the first type V secretion systems studied in detail. Many important virulence factors belong to this family, IgA protease from *Neisseria meningitidis* [23], adhesin involved in diffuse adherence (AIDA)-I from *Escherichia coli* [24] and Pertactin from *Bordetella pertussis* [25] among them. They have very diverse functions, frequently related to pathogenesis: some are extracellular proteases or lipases, while others, such as AIDA-I, are adhesins. Especially the monomeric autotransporters that harbour enzymatic activities are frequently proteolytically processed to release their passenger domains into the medium after autotransport is completed. The first autoproteolytic type Va secretion system, Tsh, was identified in avian-pathogenic *E. coli* strains [26]. This autoproteolysis is mediated by conserved residues in the autotransporter pore and a conserved cleavage site in the C-terminus of the passenger domain [27,28].

Monomeric autotransporters are expressed as a single polypeptide that contains an N-terminal signal peptide, and the proteins are secreted by the Sec machinery into the periplasm. The mechanisms of protein expression and signal peptide recognition are well established and will not be discussed in detail—it is sufficient to say that autotransporters do not behave differently from any other protein until they reach the Sec machinery. But from there, our knowledge on the mechanism of autotransport becomes more diffuse. Most findings described later were generated with single exemplars of autotransporters in a few organisms only. While the authors of this review are convinced that all basic functions are conserved, we still cannot exclude that exceptions to the rule exist.

All autotransporters need to reach the cell surface, and it is reasonable to assume that these sometimes extremely large molecules can do so only in an unfolded state. At different steps of the transport process, mechanisms are found that inhibit premature folding. Hbp, the haemoglobin protease of *Escherichia coli*, has been shown to use the Srp (signal recognition particle) pathway of co-translational translocation through the Sec machinery; this ensures that folding cannot take place in the cytosol, as the polypeptide is exported as it is synthesized by the ribosome attached to SecYEG [29]. YidC, a known accessory factor for IM protein biogenesis, was also shown to be involved in translocation; depletion of YidC leads to periplasmic aggregates of Hbp, and generally to lower level surface expression of Hbp and another autotransporter, EspC [30].

An important difference from other Sec-dependent proteins is the fact that many (but not all) monomeric autotransporters, Hbp among them, contain significantly longer signal peptides, extended at their N-terminus [29,31]. Experiments with *E. coli* EspP shed some light on the function of this extended signal peptide: when it was exchanged for a standard (short) signal, EspP started to misfold and aggregate in the periplasm, while translocation through the IM was unaffected. Moreover, the long signal peptide seemed to slow down the Sec-dependent translocation [31]. Presumably, the slower translocation allows the protein to prepare for OM autotransport, with its N-terminus still tethered to the Sec machinery that is released only after relatively late cleavage of the conserved, extended signal peptide.

In *E. coli* at least, interactions of periplasmic chaperones with unfolded autotransporters have been shown in detail—again, this presumably inhibits premature protein folding or misfolding. For EspP, direct interactions of the unfolded protein with the chaperones SurA, Skp, the chaperone/protease DegP and the *cis*/*trans*-prolyl isomerase FkpA were demonstrated [32–34], and the interaction with SurA is also known for Hbp [35]. DegP might be involved in quality control, not only serving as a chaperone but also degrading misfolded protein, as demonstrated for the trimeric autotransporter YadA [36]; please note though, that this is a type Vc autotransporter, as discussed later. However, also in the case of Hbp, degradation by DegP was demonstrated after depletion of the IM factor YidC [30]. Moreover, it seems that the passenger domains, which are typically built from repetitive β-helices, do not easily fold autonomously, but rather stay in a metastable, unfolded state that is not prone to aggregation (at least compared with other unfolded proteins) [37]. Another interesting observation in this context is the comparatively low amount of cysteine residues and disulphide bonds in secreted proteins in general [38], and specifically in autotransporters. Vice versa, when disulphide bonds are present in recombinant passenger domains, these frequently hinder autotransport, unless DsbA (the periplasmic enzyme that catalyses their formation) is deleted from the expression strain [39].

Originally, it was thought that autotransporters were able to insert into the bacterial OM without the involvement of other factors. In recent years, however, the essential OM protein BamA (originally termed Omp85 in *N. meningitidis* and YaeT in *E. coli*) has been shown to be crucial for autotransporter biogenesis. The β-barrel assembly complex, consisting of BamA and four other accessory proteins (BamB to BamE in *E. coli*), catalyses the insertion of virtually all β-barrel OM proteins [40]. Given that all β-barrel OM proteins, with the notable exception of type I secretion system components and some bacterial toxins, are homologous (i.e. evolutionarily related [41,42]), this general mechanism should also apply to autotransporters. On the other hand, numerous β-barrels are easily able to insert into membranes *in vitro* without external factors [43], suggesting that while the Bam complex catalyses fast and efficient insertion, it might be expendable in some cases. The Bam complex recognizes a C-terminal motif in β-barrel proteins [44], and this motif had already been detected in porins [45,46] and also in autotransporters [17] before the role of the Bam complex became clear. The involvement of BamA in membrane insertion of autotransporters was only recently demonstrated experimentally: BamA can be crosslinked to EspP and to Hbp during membrane insertion [32,35], and BamA (YaeT) depletion in *E. coli* and *Shigella flexneri* impairs the biogenesis of different monomeric autotransporters such as IscA or AIDA-I [47]. The sequential binding of EspP to different lipoproteins of the Bam complex, namely BamB and BamD, after BamA binding, emphasizes the importance of the machinery in the autotransport process—as in the assembly of all other β-barrel OM proteins [48]. It has even been suggested that the Bam complex itself might act as the translocation pore, but this seems unlikely for two reasons. First, the passenger domain would have to exit from the Bam pore laterally, which would entail breaking and re-forming hydrogen bonds within the barrels of both BamA and the autotransporter. Second, the autotransporter barrel is not just a membrane anchor: it cannot be functionally replaced by other β-barrel proteins and thus plays an active role in passenger domain secretion [49].

Many different models have been put forward over time of how autotransport through the OM takes place. The original model was put forward for *Neisseria* IgA protease [50]: the authors suggested that a pore is formed in the OM by the C-terminus of the autotransporter, and that a hairpin loops out through the pore; only then, does the exported passenger domain start to fold from the C- to the N-terminus, pulling out the rest of the protein in the process. This model still holds true. Other models, as reviewed by Henderson *et al.* [22], include oligomeric forms, or passenger domains exported through the lipid membrane instead of the protein pore. Different approaches have been taken over time to show that autotransporters form a pore that is used, and later occluded, by the passenger domain. The first crystal structure of the transport domain of an autotransporter was that of *Neisseria* NalP [51]. The structure clearly demonstrated a helix that occludes the pore, but as the construct was refolded from inclusion bodies, the question of a possible refolding or crystallization artefact was raised, and the matter was only laid to rest after the first complete structure of an autotransporter, EstA of *Pseudomonas aeruginosa*, was published in 2011 [52]. The structure of an autoproteolytically processed autotransporter, EspP, displays the same features as those of NalP, although the helix in the barrel is truncated [53].

Unfortunately, crystal structures can only give a static picture of the final, folded protein. The major question for the mechanism of autotransport is whether the passenger domain passes the pore N-terminus first (i.e. head-to-tail), or whether a hairpin is formed and the passenger domain loops out through the pore tail-first. Both models have their problems; if the protein inserts N-terminus first, it is unclear how the loose end (at the very end of a sometimes hundreds of residues long unfolded polypeptide) should find its exit pore; if a hairpin is formed, it remains unclear whether there is enough space in the pore to accommodate two protein chains in parallel, and what the driving force to form the initial hairpin would be. The crystal structures only demonstrated a narrow, 12-stranded β-barrel pore. But biochemical experiments all agree with the hairpin model: a mutant version of EspP that is stalled in autotransport can be crosslinked to BamA only when in the stalled form; the identified interaction sites are in the passenger domain close to its C-terminus, and in the transport domain [32]. The passenger domains seem to have their folding core at their C-terminal end and little to no autonomous folding propensity at their N-terminus, strongly suggesting a sequence of folding from the C- to the N-terminus. This was demonstrated for Pertactin [37], for Hbp [54] and for EspP, where the rest of the passenger domain was only secreted when its C-terminal part could fold, while the same C-terminal part itself could also be exported when its folding was impaired by mutagenesis [55]. This elegant experiment strongly suggests that the energy of initial folding drives the continuation of the transport process. The short autochaperone region between passenger domain and translocation domain is essential for the autotransport process, as it mediates or initiates folding of the passenger domain [56]; when it is deleted, the passenger is still exported but is protease-sensitive and thus at least partially unfolded. This again speaks for a folding mechanism that proceeds from the C- to the N-terminus. Finally, cysteine scanning mutations, again in Pertactin, showed that the C-terminal part of the passenger domain passes the autotransporter pore first, and can be physically crosslinked to the pore lumen [57].

**Figure 2.**
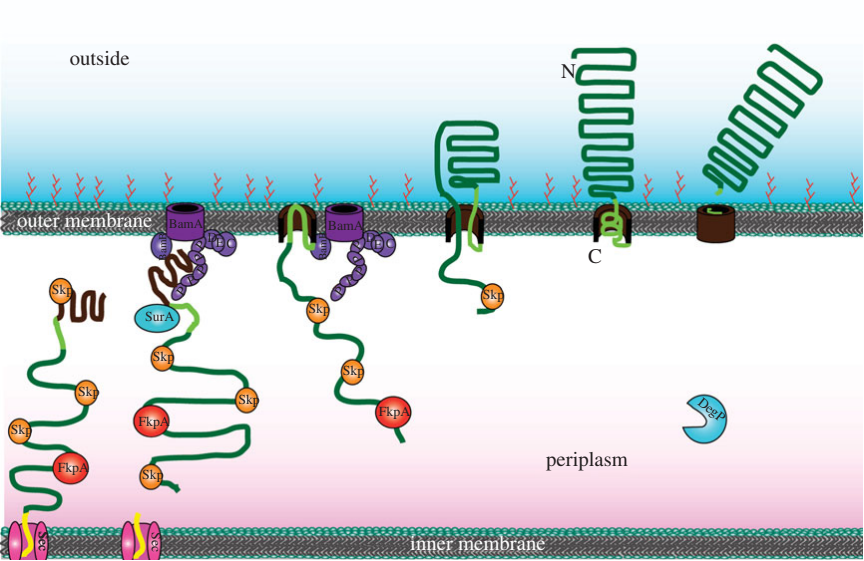
Type Va autotransport model. The autotransporter polypeptide is threaded through the inner membrane (IM) by the Sec machinery (in magenta). Many autotransporters, including the one pictured, have an extended signal peptide (in yellow) which remains attached to the Sec translocon and tethers the autotransporter to the translocon. In the periplasm, chaperone proteins such as Skp (orange), FkpA (red) and SurA (in blue) and DegP (in blue) bind to the autotransporter and keep it unfolded. The chaperone/protease DegP is also involved in quality control of autotransport. The signal peptide is eventually cleaved by signal peptidase (not shown) releasing the autotransporter into the periplasm. The C-terminal membrane anchor (in brown) is recognized by the POTRA (P) domains of BamA (in purple); the Bam complex aids in inserting the β-barrel membrane anchor into the outer membrane (OM). The linker region (light green) then forms a hairpin inside the pore of the barrel. The passenger domain (dark green) is pulled through the pore. The energy for this presumably derives from the sequential folding of the passenger domain on the outside of the cell. Once the passenger domain has been secreted, the linker assumes an α-helical conformation and plugs the pore. For many classical autotransporters and as shown here, the linker undergoes an intra-barrel cleavage to release the passenger domain into the extracellular milieu.

The current understanding of type Va autotransport is displayed in figure 2. After Sec-dependent translocation to the periplasm, autotransporters are kept in an unfolded state by chaperones, and also by their low intrinsic folding propensity. In many exemplars an extended autotransporter signal peptide is present that slows down processing at the Sec translocon, allowing the C-terminal part of the sequence to interact with the Bam complex through a conserved β-barrel recognition motif before the N-terminus is released from the IM. The Bam complex then integrates the β-barrel into the OM, and during or very shortly after insertion, the hairpin is formed that initiates the autotransport process through the newly formed pore. After the folding core at the C-terminus of the passenger domain has passed the pore, the sequential folding from the already exported C-terminal end drives the process to completion. Many but not all type Va autotransporters are autocatalytic proteases that, in a final step, cleave off their passenger domain and release it.

## 4. Type Vb secretion: two-partner secretion systems

In contrast to classical autotransporters, the passenger and translocator functions in TPSSs are located in separate polypeptide chains. By convention, the passenger polypeptide is referred to as the TpsA protein and the β-barrel transport protein as TpsB. Well-studied examples of TPSSs are the filamentous haemagglutinin (FHA) from *Bordetella pertussis* [58], the haemolytic ShlA/B system of *Serratia marcescens* [59] and the high molecular weight adhesins HMW1 and HMW2 from *Haemophilus influenza* [60]. FHA is transported through the OM by its TpsB protein FhaC, and ShlA by ShlB, whereas HMW1B and HMW2B are responsible for the transport of HMW1 and HMW2, respectively.

**Figure 3.**
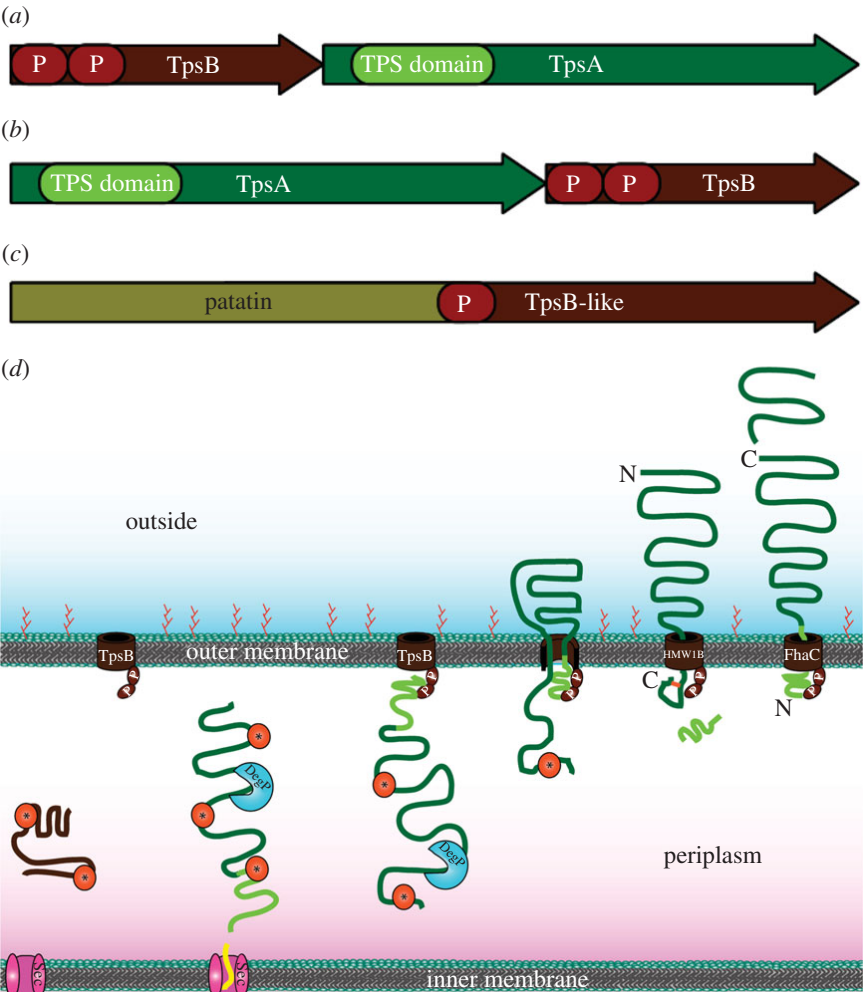
Type Vb autotransport model and genomic organization. (*a*) The genes encoding TpsA and TpsB proteins are organized into operons, where the TpsB gene usually precedes the TpsA gene. (*b*) Genomic context analysis by GCView [61] shows that the order can be reversed. (*c*) Passenger domains can be fused to their transport domains in type Vd autotransporters. TpsA proteins contain TPS domains that are responsible for the specific recognition by their cognate TpsB proteins. All TpsB proteins contain POTRA domains (see text). (*d*) The translocator TpsB protein (brown) and the passenger TpsA protein (green) are synthesized as separate polypeptide chains, which are then transported across the IM by the Sec machinery (magenta). The TpsB protein folds into a β-barrel structure in the OM, with two periplasmic POTRA domains (P). Like many classical autotransporters, some TpsA proteins such as FHA and HMW1 contain extended signal peptides (shown in yellow). Periplasmic transit of both proteins is presumably facilitated by chaperones (orange, with asterisk). In the case of FHA, DegP acts as a chaperone. The N-terminal TPS domain (light green) targets the TpsA protein to its cognate TpsB partner. The TpsA protein is then translocated across the OM into the extracellular space. The final topologies of HMW1 and FHA differ, and the alternative conformations strongly speak for a hairpin as the transport intermediate: mature HMW1 (second structure from the left) has its C-terminus locked in the periplasm by a disulphide bond (orange) that prevents its passage through the pore of HMW1B, its TpsB partner. The N-terminal TPS domain of HMW1 is cleaved, probably in the periplasm, to produce the mature passenger (dark green). FHA remains associated with FhaC through its N-terminal TPS domain, with the C-terminus distal from the cell surface, and is cleaved or partially degraded. The C-terminus of FHA is processed at the cell surface by the autotransporter protease SphB1 [62], but for clarity, in this schematic the cleaved C-terminus is shown distally from the cell surface.

The genes encoding TpsA and TpsB proteins are organized into operons, where the TpsB gene usually precedes the TpsA gene (figure 3*a*). However, genomic context analysis by GCView [61] shows that in some cases, the order is reversed (figure 3*a*). Although it would in principle be conceivable that a single TpsB was responsible for the transportation of several different TpsA proteins, this does not appear to be the case; rather, each TpsB appears dedicated to transporting only its cognate TpsA partner [63]. A notable exception is FhaC of *Bordetella bronchiseptica* that transports two different substrate proteins [64].

TpsB proteins are 16-stranded β-barrels homologous to BamA and contain two periplasmic polypeptide transport-associated (POTRA) domains. TpsA proteins are more diverse, but almost all are predicted to fold into largely β-helical structures [65]. Like many autotransporters, large TpsA proteins contain an extended signal peptide [66]. The N-terminus of TpsAs contains a highly conserved, approximately 300 residue domain, designated the two-partner secretion (TPS) domain [67]. This domain contains the targeting signal recognized by the TpsB protein, and the POTRA domains of TpsB interact directly with the domain [68]. For the interaction, the TPS domain must be unfolded [69]. However, the isolated TPS domains of HMW1 and FHA form stable, β-helical structures, but presumably do so only after secretion *in vivo* [70,71]. It is interesting to note that for *Serratia* ShlB, mutations in one of the POTRA domains did not impair translocation, but inhibited the maturation of the secreted enzyme [72].

The mechanism of transport of TpsA proteins across the OM is assumed to be similar to autotransport. The transport is a two-step process, with a periplasmic intermediate [73]. For FHA, DegP acts as a chaperone during transit [74]. The TpsA protein must remain in a transport-compatible conformation, as C-terminal fusion to the globular, disulphide-containing subunit B of cholera toxin inhibited secretion [75]. After the TPS domain has become attached to the TpsB POTRA domains, the TpsA protein is threaded through the pore of the TpsB. As the C-terminus of FHA is exposed at the cell surface, Mazar & Cotter have suggested a model in which the N-terminus of the protein remains tethered to FhaC, while the rest of FHA is exported with an N-to-C polarity. This strongly implies the formation of a hairpin within the pore, similar to autotransporters [76]; there are indications from other TPSSs that this is a general mechanism [77]. An extracellular loop (L6) of FhaC is necessary for secretion, and may be involved in initial formation of the hairpin [78]. Once the hairpin is formed, folding of the β-helix would provide the pull to bring the rest of the protein through. FHA remains tethered to FhaC for a time before being released into the extracellular medium; the C-terminus of FHA is proteolytically processed and degraded during secretion [76]. The helix formed by the N-terminus of FhaC seen in the crystal structure probably only inserts into the FhaC pore to plug it after FHA release [78].

In contrast, the final topology of HMW1 differs from FHA in that the C-terminus remains in the periplasm. The C-terminus contains a disulphide-bonded loop that prevents its passage through HMW1B [79]. The N-terminal TPS domain of HMW1 is cleaved to yield the mature protein [80], but it remains unclear whether this cleavage occurs in the periplasm or extracellularly. A model has been proposed in which HMW1 is threaded through HMW1B N-terminus first, with the TPS domain being cleaved on the outside of the cell during export [77]. However, as it is highly likely that the mechanism of secretion is conserved between type Vb systems, we favour a model in which the TPS domain of HMW1 is degraded in the periplasm [80]. In this model, the TPS domain binds to HMW1B, and most of HMW1 is exported N-to-C by the hairpin mechanism [77]. The transport is stalled at the disulphide-bonded C-terminus, and the TPS domain is consequently cleaved to release the N-terminus into the extracellular space. This model can be reconciled with the finding that cleavage of the TPS domain is not required for secretion [80] as the binding of the TPS domain to TpsB POTRA domains is reversible [68]. The stalling of the transport by the C-terminus would thus eventually lead to the dissociation of the TPS domain and its transport into the extracellular milieu via the hairpin.

The steps in TPS secretion outlined here, including the different final topologies of FHA and HMW1, are represented in figure 3*d*.

## 5. Type Vc secretion: trimeric autotransporters

Trimeric autotransporters are important virulence factors in many Gram-negative pathogens. In contrast to many of their monomeric counterparts (type Va autotransporters), they are usually adhesins, do not harbour enzymatic functions and are not released from the cell surface by an autoproteolytic mechanism. Instead, they protrude from the cell surface as relatively rigid rods, with a length of over 250 nm in some cases [81]. Trimeric autotransporter adhesins (TAAs) have a modular and repetitive architecture, and can be composed of numerous different domains. The common principle of all building blocks is their ability to trimerize, and to connect to the omnipresent stretches of trimeric coiled coils that are one of their dominant features [82]. The modular arrangement of domains presumably allows pathogens to frequently and easily recombine their adhesin repertoire [83]. But while the surface-localized part of TAAs is highly variable, the domain that really defines the family is their translocation domain, or membrane anchor [81].

The prototype of all TAAs is YadA, the *Yersinia* adhesin A of *Yersinia enterocolitica* [84], which is known to bind to collagen of the host extracellular matrix [85]. It is comparably simple in architecture, consisting only of a β-roll head domain [86] that hosts the adhesive function, an extended coiled-coil stalk and the membrane anchor [87]. Other well-studied members of the TAA family, but with a more complex domain architecture, are the *Bartonella henselae* adhesin BadA [88], the *Haemophilus influenzae* adhesins Hia [89] and Hsf [90], and the *Moraxella catharralis* ubiquitous surface proteins UspA1 and UspA2 [84,91].

Trimeric autotransporters follow the same route as type Va autotransporters for their biogenesis. They frequently have the N-terminally extended autotransporter signal peptides described earlier [82] that presumably tether the nascent polypeptide chain to the IM until Sec-mediated translocation into the periplasm is complete. There is recent evidence that periplasmic chaperones may aid in keeping YadA in an unfolded state in the periplasm. The periplasmic protease and chaperone DegP has been shown to be involved in the quality control of trimeric autotransporters: YadA is degraded when mutations are introduced that hinder efficient autotransport [36]. TAAs have little autonomous folding propensity over wide stretches of their passenger domain sequences, displayed—for example—by the need for stable, trimeric domains fused to recombinantly expressed TAA domains from the *Salmonella* adhesin SadA, which otherwise would not fold and crystallize [92]. In addition, many exemplars, including SadA [93], EibD of *E. coli* [94] and UspA1 [91], are partially composed of atypical trimeric coiled-coil domains that contain hydrophilic core residues and bind ions in their core, where usually hydrophobic interactions should stabilize the structure. These hydrophilic residues, typically asparagines, significantly reduce the folding propensity of the proteins, but keep them highly soluble as it is usually hydrophobic interactions that initiate protein aggregation. It has been speculated that this helps TAAs to maintain a transport-competent, unfolded but non-aggregated state during their passage through the periplasm and during autotransport [93].

TAAs have a β-barrel translocation domain that resides in the OM. In contrast to all other type V secretion systems, this translocation pore is not made from one, but from three polypeptide chains, where each chain contributes four β-strands to form a 12-stranded β-barrel [81,84,87]. For YadA, it was demonstrated that again, the Bam complex plays an important role in its biogenesis: depletion of BamA lead to reduced surface expression of YadA, and the C-terminal motif for Bam recognition is present in YadA [95] and all other type Vc autotransporters.

The crystal structure of the transmembrane domain of *Haemophilus* Hia [96] shows a β-barrel pore of similar outer diameter as that of monomeric autotransporters. It was noted earlier that the residues facing the pore lumen in TAAs all have small side-chain volumes, mostly glycines and alanines. When the most conserved glycine is mutated to bigger residues in YadA, autotransport is significantly slowed down, and the periplasmic stress response towards misfolded proteins is turned on [36]. This already demonstrates that autotransport must proceed through the pore. Moreover, the transmembrane domain of TAAs is sufficient for trimerization and export; passenger domains from different TAAs fused to the same transport domain are able to form mixed trimers in the membrane and can export mixed passenger domains to the cell surface [97]. Interestingly, the part of the sequence that later occludes the pore cannot be deleted without losing the capacity to form trimers in *Haemophilus* Hia [96], and in the case of YadA, deletion of this region abolished protein production completely [98]. This speaks for the involvement of this region in a transport intermediate, where trimerization and presumably, hairpin formation are coupled.

All passenger domains of TAAs contain a coiled-coil segment N-terminal to the membrane anchor, and many TAAs have a specific sequence motif, [YxD] in right-handed coiled-coils, or the analogous motif [RxD] in left-handed coiled coils, in this region, sometimes repeatedly. It seems to be a folding core motif [99], which would be in good agreement with a hairpin model for autotransport, where all three hairpins are exported in a concerted manner. The Y/RxD motif would then start the folding process from the C- to the N-terminus, analogous to the sequential folding in type Va autotransporters, and all three chains would be driven out and fold simultaneously, through an admittedly extremely tight pore. The previously described coiled-coil motifs that sequester ions to their core might have a comparable role in initiating folding, once they have passed the pore [93].

The transition of one handedness to another in the coiled-coil segment might alleviate torsion stress during folding [99], and the same could apply to other TAA-specific structural features, such as the neck or the saddle [94].

**Figure 4.**
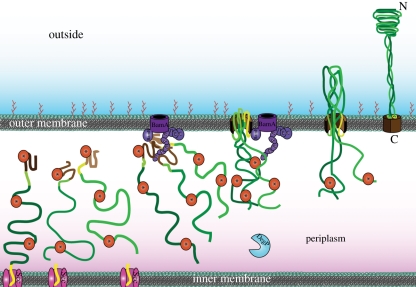
Type Vc autotransport model. Trimeric autotransport presumably follows a largely similar sequence of events to classical autotransport, the major difference being the presence of three polypeptide chains rather than just one. Many trimeric autotransporters also contain extended signal peptides, shown in yellow. As yet unidentified chaperones (orange, marked with asterisks) keep the polypeptides in a translocation-competent, unfolded state. The Bam complex (purple) is required for trimeric autotransporter biogenesis and recognizes the C-terminal membrane anchor (in brown, the three membrane anchor monomers are coloured in different shades). The Bam complex assists in trimerization of the β-barrel and membrane insertion. The linker regions (different shades of light green) form hairpins within the pore, and this leads to translocation of the polypeptides encoding the passenger domain (in different shades of dark green). The periplasmic chaperone/protease DegP is involved in quality control of trimeric autotransport. The passenger domain trimerizes after secretion and remains covalently attached to the membrane anchor.

The current model of type Vc autotransport is displayed in figure 4. After Sec-dependent translocation to the periplasm, trimeric autotransporters are kept in an unfolded state by chaperones, and also by their low intrinsic folding propensity. An extended autotransporter signal peptide is present in many exemplars and slows down processing at the Sec translocon, allowing the C-terminal part of the sequence to interact with the Bam complex through a conserved β-barrel recognition motif before the N-terminus is released from the IM. The Bam complex then mediates trimerization and integrates the β-barrel into the OM. During or very shortly after insertion, the three hairpins are formed that initiate the autotransport process through the newly formed pore. After the folding core at the C-terminus of the passenger domain has passed the pore, the sequential and concerted folding from the three already exported C-terminal ends drives the process to completion.

## 6. Type Vd secretion: fused two-partner secretion systems?

Recently, a novel type of autotransporter was described, and was termed type Vd secretion [100]. It has probably escaped attention for a long time as it is predominantly present in environmental organisms, which have only recently captured the attention of sequencing projects. The prototype of this new family is a patatin-like protein from *Pseudomonas aeruginosa*, named PlpD. Its passenger domain is a lipolytic enzyme and is cleaved autocatalytically after completion of autotransport, not unlike some lipases exported by the classical type Va pathway. But in PlpD, the passenger domain is connected to the translocator β-barrel domain with a POTRA domain (figure 3*c*). Thus, it looks like a gene fusion of the two components of a TPSS [100,101], albeit with a different type of passenger domain. For type Va autotransporters, it has been demonstrated that the passenger domain at its C-terminal end interacts with the POTRA domains of BamA, and thus that POTRA domains play a role not only in β-barrel insertion, but probably also in initiating autotransport [48]. It is conceivable that in PlpD this function is fulfilled by the intrinsic POTRA domain, especially as in two-partner secretion the POTRA domains in TpsB interact directly with the TPS domain responsible for recognition and export of TpsA [68].

The case of PlpD is interesting in two ways: first, it demonstrates that more classes of unrecognized autotransporters might exist, either because they are not similar enough to the known types and thus not easily found with BLAST in sequenced genomes, or because they are present in groups of organisms underrepresented in the databases (e.g. because they are non-pathogenic). Second, PlpD clearly links type Va and type Vb (two-partner) secretion, and thus puts an end to the debate on whether TPSSs are really autotransporters or not [102,103]. Even TPSS gene fusions with typical (i.e. β-helical) passenger domains can be found in the database, such as Tery_3487 of *Trichodesmium erythraeum* [101].

## 7. Type Ve secretion: classical autotransport, but inverted?

Intimin of *E. coli* and Invasin of enteropathogenic *Yersinia* spp*.* are closely related adhesins with extracellular Ig domains anchored in the bacterial OM—related enough to allow for a chimeric exchange of their transmembrane domain, which still yields functional protein [104]. Intimin mediates the intimate attachment of pathogenic *E. coli* to host cells, which leads to pedestal formation. To this end, a second protein (Tir) is secreted into the host cell and is inserted into the host cell plasma membrane from the inside [105]. The secretion and injection of Tir is mediated by a type III secretion system. Tir in the host cell membrane then acts as the specific receptor for the binding domain of Intimin [106]. Invasin, in contrast, directly binds to β_1_-integrins that are located on the surface of the host cell [107]—no other bacterial factor, and no type III secretion system, is required.

Both proteins are well studied for their involvement in pathogenesis. In contrast, numerous models only vaguely describe the membrane anchor of these proteins as a transmembrane β-barrel. They avoid—probably deliberately—a clear discussion on the topology and connectivity between the extracellular domains and the anchor, albeit suggesting that they are autotransporters [108–111]. Even the fact that Intimin can be used for surface display of heterologous proteins [108] just like in the autodisplay expression systems based on classical type Va autotransporters [19] did not lead to the label ‘autotransporter’ for these proteins.

Only recently, a detailed topology model was published for the Intimin/Invasin family [112], showing a small, N-terminal periplasmic domain (that probably binds peptidoglycan [109]), followed by a transmembrane β-barrel domain. This domain forms a pore that is occluded by an α-helix, which in turn connects the pore to the exported Ig domains (figure 1). Even though, in the light of the literature cited earlier, we disagree with the authors that the Intimin/Invasin family of proteins is ‘novel’ [112], our own bioinformatics and experimental data strongly support their topology model and thus the notion that we are dealing with an autotransport system. The experimental support includes the insertion of immunogenic tags into different positions of the protein, and experiments that show their localization in the periplasm or on the cell surface, respectively [113].

**Figure 5.**
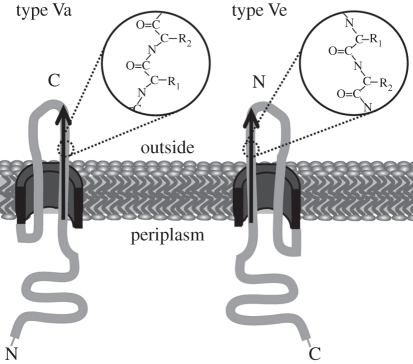
Type Ve autotransport differs from type Va. Autotransport proceeds from the N- to the C-terminus in type Ve autotransporters such as Intimin and Invasin (from the C- to the N-terminus in type Va autotransporters). For emphasis, a short section of the polypeptide chain is shown, where R_1_ and R_2_ are side-chains, and R_1_ is N-terminal to R_2_.

In contrast to type Va autotransporters, the order of passenger and transport domains are reversed in Intimin and Invasin. Thus, it is the C-terminus, not the N-terminus, that extends from the OM to the host cell. This fact must consequently mean that the mechanism of autotransport is different from type Va autotransport, as a presumed hairpin intermediate would loop through the transport pore pulling a strand in the inverse direction and the exported domain would fold from its N- to its C-terminus (not C- to N-terminus as in type Va autotransporters, figure 5). We propose here that this system should be labelled ‘type Ve secretion’, to acknowledge this significant difference from type Va secretion.

Apart from the inverted transport mechanism through their OM pore, Intimin, Invasin and their homologues seem to follow the same export route as classical autotransporters. Intimin has a long Sec signal peptide [109] and thus is translocated by the Sec machinery into the periplasm, as is Invasin. The passenger domain of Intimin is kept in a translocation-competent (i.e. unfolded) conformation in the periplasm [114], and Intimin insertion into the OM depends on BamA and the chaperones SurA and Skp, and on DegP that plays a role in quality control and degradation of misfolded variants [115].

## 8. Conclusions and outlook

There is an established evolutionary relationship between the transport domains of autotransporters and almost all other OM β-barrel proteins [42,116]. Thus, it is not surprising that they use the same OM insertion machinery (the Bam complex) and contain the same insertion signal at the C-terminus of their β-barrel domain, as discussed already. The involvement of the same IM translocation machinery (the Sec translocon), the same set of periplasmic chaperones (such as Skp and SurA), of prolyl-*cis*/*trans* isomerases (such as FkpA) and of quality control proteases (such as DegP) for classical β-barrel proteins [117,118] and autotransporters (see §3) strongly suggests that there is a general mechanism for the secretion of all β-barrel proteins, including all autotransporter classes.

The difference is the autotransport itself, an event that we strongly believe starts after, or at most synchronously, with OM insertion, and thus represents an addition to the general OM insertion mechanism mediated by the Bam complex. It has been suggested that folded or semi-folded periplasmic intermediates of autotransporters exist. The experiments that lead to this conclusion [32,35,119] are not necessarily a contradiction to our view, but probably demonstrate a synchronization of membrane insertion and autotransport initiation. In detail, Ieva *et al.* [119] demonstrate that in a mutant stalled in autotransport, a protease-insensitive intermediate exists that can be extracted using urea. Thus, it cannot be fully inserted in the membrane at this stage. But a fully folded transport domain as well as the notion of other pre-folded β-barrel proteins [120] in the periplasm before membrane insertion is unlikely. There is no system to transfer energy to the Bam complex from the IM. Thus, the energy for membrane insertion must stem from the formation of the many hydrogen bonds formed during β-barrel protein folding: insertion and folding must be coupled. A preformed structure would have to either unfold again, or to insert in folded form, a bit like the cork being pushed back into a champagne bottle. Both ‘mechanisms’ would need considerable amounts of energy, not available in the periplasm.

The same energy considerations hold true for autotransport as such (i.e. the events after membrane insertion). Neither ATP nor a proton gradient is necessary for surface display of the transported passenger domains. Again, the only source of energy for transport available is the free energy of protein folding. Key experiments have recently substantiated this view, showing that folding of type Va autotransporters proceeds sequentially from the C- to the N-terminus, driving the process further [37,55]. The same experiments, and others reviewed earlier, also clearly speak for a hairpin that is formed to initiate the autotransport, illustrated in figures 2–5 for the different autotransporter subtypes. In this context, it is also interesting to note that in TPSSs (type Vb secretion), the exported domain can stay attached to the transport domain either with its N- or its C-terminus (see §4). As the transport domains do not differ significantly, a common transport mechanism can be assumed: only a hairpin intermediate explains these alternative conformations satisfactorily.

The major objection raised against the hairpin model of autotransport is the restricted size of the β-barrel pore. The crystal structures of the translocation domains of NalP and other type Va systems and of the type Vc system Hia in principle show that there is enough room for two extended polypeptide strands in the pore (six in the case of type Vc), as discussed in [36,51,96]. Moreover, β-barrel proteins are not static structures, but capable of undergoing significant conformational fluctuations without breaking down the hydrogen bonds that define the barrel. This is not only observed in electrophysiological measurements, but was also recently demonstrated for the usher β-barrel protein FimD, which, when co-crystallized with its transport substrate, had a significantly wider pore size compared with the apo-structure [121]. It is thus also possible that autotransporter β-barrels are capable of such temporary expansions during transport, even though such fluctuations were not observed in molecular dynamics simulations of NalP [122]. This is probably due to the short timescale of the simulations (10 ns), and due to the fact that the final structure, and not a model of a transport intermediate, was used.

We conclude from the literature compiled in this review that there is a general mechanism for autotransport, where the different types of autotransporters follow the general route for β-barrel protein insertion into the OM. An extended signal peptide in many cases ensures slow processing by the Sec machinery, to gain time for proper OM insertion before the passenger domain is released. Moreover, premature folding in the periplasm is inhibited by the known periplasmic chaperone systems, and also by sequence-intrinsic properties of the passenger polypeptides, such as a reduced folding rate, little to no cysteine residues for disulphide formation, high solubility of the unfolded passenger domains and little to no propensity to aggregate when in the unfolded state. Most probably already during membrane insertion a hairpin structure is formed, and the sequential folding of the passenger domain on the cell surface drives the process to completion.
